# Oral contraceptive pills shortage in Lebanon amidst the economic collapse: a nationwide exploratory study

**DOI:** 10.1186/s12913-023-09523-3

**Published:** 2023-05-23

**Authors:** Rania Itani, Hani MJ Khojah, Samar Karout, Rana Abu-Farha, Tareq L. Mukattash, Deema Rahme, Khouloud Housary, Hiam El Achi, Ali O. Safar, Ismail Karam Al Hajj, Abdalla El-Lakany

**Affiliations:** 1 Pharmacy Practice Department, Faculty of Pharmacy, Beirut Arab University, Riad El Solh, P.O. Box: 11-5020, Beirut, 1107 2809 Lebanon; 2 Department of Clinical and Hospital Pharmacy, College of Pharmacy, Taibah University, P.O. Box: 30051, Madinah, 41477 Kingdom of Saudi Arabia; 3 Department of Clinical Pharmacy and Therapeutics, Faculty of Pharmacy, Applied Science Private University, P.O. Box: 11931, Amman, Jordan; 4 Department of Clinical Pharmacy, Faculty of Pharmacy, Jordan University of Science and Technology, P.O. Box: 3030, Irbid, 22110 Jordan; 5 Obstetrics and Gynecology Department, Faculty of Medicine, Beirut Arab University, Riad El Solh, P.O. Box: 11-5020, Beirut, 1107 2809 Lebanon; 6 Internal Medicine Department, Faculty of Medicine, Beirut Arab University, Riad El Solh, P.O. Box: 11-5020, Beirut, 1107 2809 Lebanon; 7 Department of Pharmaceutical Sciences, Faculty of Pharmacy, Beirut Arab University, Riad El Solh, P.O. Box: 11-5020, Beirut, 1107 2809 Lebanon; 8 Department of Pharmacognosy, Faculty of Pharmacy, Alexandria University, Alexandria, Egypt

**Keywords:** Oral contraceptive pills, Contraception, Economic crisis, Women, Reproductive health, Abortion, Lebanon

## Abstract

**Background:**

The political instability, economic crisis, and devaluation of the national currency left Lebanese females suffering from a scarcity of oral contraceptive pills (OCPs). Therefore, we aimed to identify the incidence of OCPs shortage in Lebanon and its impact on women’s sexual and reproductive health, as well as physical and psychological well-being.

**Methods:**

Community pharmacies were selected randomly across Lebanon, using a stratified sampling approach, where female clients asking for OCPs were interviewed using a standardized data collection form.

**Results:**

A total of 440 females were interviewed. More than three-quarters of the participants (76.4%) reported not finding their preferred OCPs brands, almost 40% were affected by the increased prices, and 28.4% declared stockpiling OCPs. More than half of the participants using OCPs for pregnancy prevention reported adopting alternative traditional contraceptive methods (55.3%). Unplanned pregnancy was reported by 9.5% of participants, where 75% of them disclosed intentional abortion while the remaining (25%) reported experiencing a spontaneous miscarriage. Other consequences of OCPs shortage included mood disturbances (52.3%), dysregulation of menses (49.7%), dysmenorrhea (21.1%), weight gain (19.6%), acne (15.7%), and hirsutism (12.5%). Of the participants taking OCPs for birth control, 48.6% reported a reduced frequency of sexual intercourse, which led to conflicts with their partners (46%) and a decreased libido (26.7%).

**Conclusions:**

OCPs shortage has seriously and negatively exposed women to various undesirable consequences including unplanned pregnancy and dysregulation of menses. Therefore, there is an urgent need to bring the attention of healthcare authorities to support the national pharmaceutical industry in manufacturing affordable OCPs generics to meet women’s reproductive health demands.

## Background

For nearly three years, Lebanon has been assailed by a multifaceted crisis. It started with political instability that led to a financial and economic meltdown followed by a series of events during and after the COVID-19 pandemic and the Beirut port explosion [1, 2]. The Lebanese Lira has lost more than 90% of its value, where in 2022 Lebanon was set for the second-highest inflation rate globally [3]. Moreover, stringent capital control was informally adopted by the Lebanese banking sector, which restrained the depositors from withdrawing their saved money in “Fresh Dollars”. This devastating humanitarian crisis that Lebanon is witnessing has led to a dramatic collapse in the basic services, that were driven by the depletion of the foreign currency, including the difficulty in securing food, fuel, medications, and medical supply [1, 3].

After the Lebanese government announced the lifting of medicine subsidies, the prices of medications skyrocketed [4]. The situation has impacted Lebanon’s ability to import essential goods including medications. This, in turn, allowed many pharmaceutical companies to make illicit profits through rationing the distribution of medications among pharmacies, and expanded the area for smuggling and monopoly of medicines without serious controlling measures being taken by the government [5, 6]. This compounded situation has spurred an unprecedented catastrophic medication shortage, where the medication supply was depleted, and community pharmacies’ shelves were empty [4, 7].

Moreover, the COVID-19 pandemic has exponentially disrupted the delivery of healthcare services worldwide [8]. Governments of different countries have issued emergency commands pausing the delivery of health services [9]. This action also left devastating economic, humanistic, and clinical effects on stakeholders from the low, middle, and high-income countries [10]. The shortage of potentially lifesaving and necessary drugs such as chemotherapy agents, contraceptives, analgesics, injectables, dietary supplements, anesthetics, anti-infectives, and cardiovascular medications were among the most common [11, 12]. The impact of this shortage left multifaceted outcomes not only for the patient but for health care providers, influencing their practice [13, 14]. It has resulted in delayed treatment for patients, prolonged recovery, increased medical costs, medication rationing, and, in some cases, denying the treatment because of the unavailability of critically important drugs [13, 15].

Drug shortage is a major public health threat. The American Food and Drug Administration (FDA) defines drug shortage as “a situation in which the total supply of all clinically interchangeable versions of an FDA-regulated drug is inadequate to meet the current or projected demand at the patient level”. Drug shortage may often impact vulnerable populations including cancer patients, neonates, the elderly, and women. It forces healthcare providers to prescribe infrequently used medications and dosage regimens, which in turn can lead to medication errors as demonstrated by earlier studies [16, 17].

While the pandemic has impacted vulnerable populations worldwide, women have faced unique challenges, particularly in low and middle-income countries. When health care systems faltered, inequalities were aggravated, and women’s specific needs became deprioritized. Hence, they faced additional barriers to care, particularly in receiving sexual and reproductive health care [18, 19]. For example, around 151 million women aged 15–49 worldwide were using contraceptive pills as their method of choice for birth control in 2019. The associated shortage of these pills in low-income countries during the COVID-19 pandemic has dramatically impacted women’s quality of life [18]. In addition, contraceptive hormone pills are commonly used for the treatment of dysmenorrhea worldwide [20, 21]. Therefore, their shortage has another negative impact on women’s health.

The implications of contraceptive drug shortages include unexpected pregnancies, abortion, deterioration of existing polycystic ovarian syndrome (PCOS), and endometriosis-associated pain [22–25]. Therefore, alternative approaches to tackle the shortage should be warranted to mitigate the clinical consequences of these shortages [26]. However, although an alternative contraceptive drug could be used, it is a frustrating task for women who have been in a ‘trial and error’ with a variety of contraceptive pills to find out the most appropriate and effective contraceptive pill [27, 28]. It is also worth mentioning the cost, particularly amidst the economic turmoil resulting from the COVID-19 pandemic, where many women lost their jobs, hence, switching to more expensive pills may affect their compliance. Furthermore, in women with endometriosis and PCOS, certain drug regimens are clinically preferred so that shortages may compromise their quality of life. For instance, progestin-only pills may be a better first-line treatment for endometriosis than combined estrogen-progestin pills. The shortage of the former pills may lead to insufficient therapeutic response when women must use the latter available pills [27, 28].

Addressing drug shortages remains a top priority for the FDA, and manufacturers and other stakeholders also have important roles to play in ensuring that critical drugs remain available for patient care [13]. In the wake of COVID-19, particularly in the developing world, evidence of the impact of contraceptive drug shortage on women’s health remains unknown.

In Lebanon, the scenario was unique because several factors, other than the pandemic, have contributed to the aggravation of the drug shortage including contraceptives. These include a shortage in fuel that affected the transportation of medicines to retail pharmacies, the reliance of pharmaceutical companies on imported drugs, the Beirut Port blast that halted at least one year of drug supply, currency inflation, poor availability of local manufacturing, patient stockpiling, cross border smuggling, and the absence of regularity procedures [29, 30]. As a result, pharmacies ran out of stock of almost all brands of contraceptive pills, leaving women without alternatives but to seek help from non-governmental organizations (NGOs) or to buy them from neighboring countries [29, 31]. Moreover, the shortage of OCPs in Lebanon may also have other negative consequences. In previous studies, some female university students have declared using OCPs as a protection method against conception outside marriage [32, 33]. The shortage may lead to unexpected pregnancies, especially when condomless sex is practiced.

With all the aforementioned implications of OCPs shortage in Lebanon, the main objectives of this study were: [1] to identify the incidence of OCPs shortage and the response strategies to address it; [2] to illustrate the impact of OCPs shortage on women’s sexual and reproductive health, as well as physical and psychological well-being; and [3] to explore the impact of OCPs increased prices and their affordability. The findings may provide critical information for drug agencies, manufacturers, stakeholders, and local and international humanitarian actors to promptly intervene in addressing the contraceptive shortage in countries with economic despair and crisis.

## Methods

### Study design

This exploratory, descriptive, and cross-sectional study included structured face-to-face interviews with adult women of reproductive age (18–49 years) receiving oral contraceptive pills (OCPs) and residing in Lebanon. The study was conducted between October 2021 and February 2022, when Lebanon was experiencing the most disastrous shortage of vital medications, concomitantly with the COVID-19 pandemic and the economic crisis, as well as the lifting of medications subsidies that led to an exponential increase of the drug prices [1–4, 7, 34, 35].

### Study setting and sampling technique

A random sample of community pharmacies in Lebanon was selected for interviewing the study subjects because these pharmacies are the main source for obtaining the OCPs. These pharmacies are independent, privately owned retail pharmacies, and are the only authorized source for dispensing medications to the public [36]. Currently, there are 2,680 community pharmacies actively operating all over Lebanon [37]. Therefore, to obtain a representative sample of pharmacies, a stratified sampling technique was employed, in which six strata were formed based on the division of the Lebanese governorates. The distribution of pharmacies among the governorates is as follows: Beirut (236, 8.9%), Bekaa (297, 11.1%), Mount Lebanon (1,114, 41.6%), North Lebanon (365, 13.6%), South Lebanon (395, 14.7%), and Nabatieh (273, 10.1%). A comprehensive list of pharmacies was retrieved from the Order of Pharmacists of Lebanon (OPL) including location, assigned governorate and district, and phone number [37]. The list was then entered into an excel spreadsheet, organized as per the allocated governorate, and each pharmacy was given a code. Within each stratum, a random sample of pharmacies was selected so that the number of designated pharmacies from each stratum is proportionate to the total number of pharmacies in that stratum. This would likely ensure a homogenous and uniform presentation of all pharmacies from all Lebanese governorates.

### Sample size calculation

The online Raosoft® calculator was used to compute the sample size of pharmacies encountering females who request contraceptive pills on daily basis for the current study [38]. A conservative assumption that results in the largest sample size was adopted. Therefore, the response distribution was set to 50% (i.e., it is assumed that 50% of Lebanese pharmacies are encountering females requesting contraceptive pills on daily basis). The sample size of pharmacies was calculated at a 95% confidence interval using an absolute precision (i.e., margin of error) of 10%, and a design effect of 1. Thus, the calculated sample size for the community pharmacies was 93. As such, to achieve a representative sample using a stratified sampling technique, the distribution of the community pharmacies that were randomly selected was as follows: Beirut (9, 8.9%), Mount Lebanon (38, 41.6%), South Lebanon (14, 14.7%), Bekaa (11, 11.1%), North Lebanon (12, 13.6%), and Nabatieh (9, 10.1%).

Afterward, the pharmacy owners of the randomly selected community pharmacies were phone called by the study principal investigator (PI) to explain the study purpose, assure the anonymity of the retrieved data, and invite them to participate. The pharmacy owners were asked to host data collectors, senior medical students, at their pharmacies to interview females asking for OCPs. A pharmacy was replaced with the next one from the remaining shuffled list, within the same governorate, whenever the pharmacy owner refused to participate in the study or when the call was not answered upon two spaced attempts during the study period. The number of clients asking for contraceptive pills varies in each pharmacy. Therefore, we decided to interview 5 female clients in each pharmacy to ensure an equal distribution of participants in each pharmacy. Hence, 465 women asking for OCPs were interviewed throughout the study.

### Questionnaire development

A standard questionnaire form was developed by the research team to tackle the targeted study objectives. The questionnaire consisted of six main sections, with 45 close-ended questions (with pre-specified answers) and open-ended questions to record the exact participants’ verbatim. The participants’ verbatim were translated into English and quoted in the study results without performing qualitative thematic analysis.

The first section was designed to gather sociodemographic information of the study subjects. The second section recorded the brand name of the utilized OCPs, indication, mode of administration, duration of use, whether the patient had already stockpiled her OCP brand, and if she is able to find it in the Lebanese market. The third section addressed the measures taken by the participants experiencing the OCP shortage. The fourth section documented the impact of this shortage on the participants’ psychological and physiological health. In this section, the participants were asked to grade their level of stress when they did not find their OCPs, measured on a five-point Likert scale (1 = totally unstressed, 2 = unstressed, 3 = neutral, 4 = stressed, and 5 = totally stressed). The fifth section assessed the impact of the increased prices of OCPs on females’ adherence. Finally, the last section was only concerned with females receiving OCPs as a birth control method. In this section, females were inquired about the reasons to avoid pregnancy, alternative contraceptive methods adopted during the shortage, measures that will be taken in case of unplanned pregnancy, and the influence of this shortage on women’s sexual life.

### Questionnaire revision and pilot testing

The questionnaire was reviewed for face and content validity by an expert panel in the field. Five experts in social pharmacy, pharmacy practice, public health, as well as community pharmacists, were asked to assess the clarity, understandability, and organization of the constructed questionnaire. To determine the content validity of the developed questionnaire, the Item Objective Congruence (IOC) index was utilized. The experts were inquired to score the quality of the questionnaire items using the IOC score method (score = 1 if the expert is sure that this item measured the attribute, score = 0 if the expert is not sure that the item does measure or does not measure the expected attribute, and score = -1 if the expert is sure that this item does not measure the attribute). The qualified items yielded an IOC score of 0.74, thus the questionnaire has acceptable content validity.

The questionnaire was then translated from English to Arabic, the national native language in Lebanon. The translation was verified by the back-and-forth method by two bilingual authors (RI and HMJK) who have academic backgrounds and experience in the provision of pharmaceutical services.

Afterward, the questionnaire was piloted on ten females known to take OCPs, chosen by a convenience approach. The participants were asked to evaluate the structure of the questionnaire, length, clarity, and give an overall impression. A minor amendment was then made based on their suggestions. Two weeks later, the questionnaire was retested on the same participants to ensure its reliability and reproducibility. The data obtained from the pilot test was not included in the final data analysis.

### Data collectors training

The data collectors were extensively trained by the PI to adequately interview participants and document the responses using the data collection form. Several role-plays were performed on different occasions between data collectors and another study researcher, acting as a mock patient receiving OCPs. Meanwhile, the PI was observing and assessing the performance of the data collectors during the role-plays and giving her feedback. The data collectors and the PI documented the mock participant’s responses, using the data collection form, and compared their findings to ensure the consistency of the recorded observations. The data collectors were instructed to adhere to the agreed introductory script and to maintain neutrality at all times in order not to influence the participants’ responses.

Afterward, before the commencement of the study, the protocol was tested on 10 pharmacies aside from the randomly selected ones. Each data collector, accompanied by the PI, visited one community pharmacy and interviewed two participants. This aided in testing the interviewing skills of the data collectors, as well as the clarity and feasibility of the questions.

### Data collection

Two data collectors were assigned for each governorate. They approached the encountered females, explained the purpose of the study, and assured their voluntary participation and anonymity. Female clients accompanying a friend, a family member, or a husband were not approached to avoid their response being influenced by the presence of their company. Finally, face-to-face interviews were conducted with the participants, and their responses were directly documented using the paper questionnaire. The average time spent per interview was 10–15 min.

### Ethical considerations

The World Medical Association Declaration of Helsinki guidance was followed in designing and conducting this study [39]. The protocol was also approved by the Institutional Review Board of Beirut Arab University (No. 2022-H-0081-P-R-0472). The purpose of the study was clearly stated to the pharmacy owners as well as the female participants. The participants had the right to defer from continuing the interview at any time. An informed consent was obtained from all the study subjects. The details related to the pharmacy or the participants’ identification were coded. Participants were not contacted afterward for any additional questioning.

### Data preparation and analysis

Responses were screened for completeness, legibility, and clarity of the participants’ verbatim. The dataset of valid questionnaires was entered and coded in an electronic spreadsheet. Then the data was reviewed and cross-checked with the original records. Data were analyzed using the 24th version of the Statistical Package for the Social Science (SPSS®, IBM Corp., Armonk, NY, USA). Descriptive data were represented by frequencies and percentages (for categorical variables) and mean with standard deviation (for continuous variables).

## Results

### Participants’ sociodemographic data

Of the total 93 contacted pharmacy owners, 9 (9.6%) refused to participate in the study, and they were replaced with others from the shuffled list within the same governorates. The study included 440 valid client responses out of 465 (Table 1). Around half of the participants were married (237, 53.9%), and almost one-fourth of them had one or two children (115, 26.2%).

**Table 1:** Participants’ Sociodemographic Data (N = 440)

Information	n (%)
***Age (mean ± SD)***	29.37 ± 8.61
18–25	198 (45)
26–35	123 (28)
36–45	96 (21.8)
> 45	23 (5.2)
***Nationality***	
Lebanese	400 (90.9)
Syrian	18 (4.1)
Palestinian	20 (4.5)
Iraqi	2 (0.5)
***Marital status***	
Married	237 (53.9)
Single	196 (44.5)
Divorced/widowed	7 (1.6)
***Number of children (range*** *)*	0–6
No children 1–2 3–4 5–6	235 (53.4) 115 (26.2) 79 (17.9) 11 (2.5)
***Educational level***	
Uneducated	53 (12)
School	40 (9.1)
Graduate studies	270 (61.4)
Postgraduate studies	77 (17.5)
***Monthly household income***	
Preferred not to say	87 (19.8)
< $250	208 (47.3)
$250–$500	97 (22)
$501–$1,000	23 (5.2)
$1,001–$2,000	14 (3.2)
> $2,000	11 (2.5)

***SD***, standard deviation

### OCPs used by the participants and their availability in the market

The study participants reported using 13 different OCP brands. Most participants were using combined oral contraceptives (398, 90.45%) while only 42 (9.54%) were using progestogen-only pills. Around 40% of participants (177, 40.5%) reported using OCPs for contraceptive purposes.

A lower percentage of the participants reported taking OCPs for period regulation (116, 26.1%), PCOS (96, 21.8%), and primary dysmenorrhea (7, 1.6%). Other uses (37, 8.4%) included acne, hirsutism, amenorrhea, pregnancy stabilization, and postponing menstrual periods. Table 2 summarizes the preferred brands, uses, and availability in the market.

Almost one-third of the participants conveyed stockpiling their preferred OCPs during the medication shortage period (125, 28.4%). For example, “my niece is a community pharmacist, so I never had to worry about the medication shortage, since she already preserved for me several boxes of my preferred brand with a reduced public price,” Participant 253 stated. “It was very hard for me to find Yasmin®, at first I visited more than seven pharmacies in Beirut and they all did not have it, but then I found it in a pharmacy outside Beirut, which had four boxes left, and I got them all, don’t ask me how did the pharmacist accept to give me all this quantity,” Participant 91 declared.

Remarkably, three-quarters of the participants reported that their OCPs brand is not available and that they are not able to find it in the Lebanese community pharmacies (336, 76.4%). The duration of the unavailability of preferred brands in the pharmacies ranged from less than 3 months as reported by 32.7% of the participants (110) to more than 3 months as reported by the remaining participants (226, 67.3%). “It is obviously inconvenient as I have to search almost ten pharmacies in Sidon to search for my preferred brand,” Participant 93. “Aside from feeling frustrated, I am confused as to why this is happening in the first place,” Participant 12 said.

**Table 2:** Oral contraceptive pills usage and their availability in the community pharmacies (N = 440)

Item	n (%)
***Usual OCPs being used by the participants***	
Ethinylestradiol/drospirenone	209 (47.6)
Ethinylestradiol/cyproterone acetate	70 (16)
Ethinylestradiol/levonorgestrel	42 (9.6)
Ethinylestradiol/desogestrel)	41 (9.3)
Dydrogesterone	30 (6.8)
Norethindrone	25 (5.6)
Ethinylestradiol/dienogest	9 (2)
Desogestrel	7 (1.6)
Progesterone	5 (1.1)
Norgestrel/estradiol)	2 (0.4)
***Indication***	
Contraception (birth control)	177 (40.5)
Period regulation	116 (26.1)
Polycystic ovarian syndrome	96 (21.8)
Primary dysmenorrhea	7 (1.6)
Endometriosis	7 (1.6)
Others	37 (8.4)
***Duration of administration***	
Intermittently	25 (5.7)
≤ 3 months	104 (23.6)
> 3 months to ≤ 6 months	65 (14.7)
> 6 months to ≤ 1 year	72 (16.3)
> 1 year to ≤ 2 years	39 (8.9)
> 2 years to ≤ 3 years	21 (4.8)
> 3 years	114 (26)
***Stockpiling behavior of preferred brands during the shortage period***	
No	315 (71.6)
Yes	125 (28.4)
***Availability of preferred brands in the pharmacies***	
No	336 (76.4)
Yes	104 (23.6)
***Duration of unavailability of preferred brands in the pharmacies (n = 336)***	
≤ 3 months	110 (32.7)
> 3 months to 6 months	125 (37.2)
> 6 months to < 1 year	57 (17)
≥ 1 year	44 (13.1)

### Adopted measures by the participants during the OCPs shortage

Participants who reported not finding their preferred OCPs in the Lebanese community pharmacies (336, 76.4%) were inquired about the strategies adopted to cope with this shortage. Most of these participants reported visiting the pharmacies three or more times within the last month aiming to find their unavailable OCPs (233 out of 366, 69.3%). Almost one-fourth of them noted using an alternative OCPs brand (94 out of 336, 27.9%). “I’ve tried several brands for years to find the right contraceptive pill brand for me, but now I’m forced to go through the same dilemma to find a suitable alternative brand,” Participant 24 responded. “The pharmacist gave me an alternative brand for birth control. However, I’m experiencing bloating and headache,” Participant 389 answered. Check Table 3 for the measures adopted by the participants during the OCPs shortage.

**Table 3:** Adopted measures by the participants during the OCPs shortage (n = 336)

Item	n (%)^a^
Visit other pharmacies to find the unavailable preferred brand	215 (63.9)
Use alternative birth control methods	166 (49.4)
Wait for the preferred OCPs brand to become available	104 (30.9)
Get the preferred OCP brand aboard	94 (27.9)
Utilize an alternative OCPs brand	94 (27.9)
Do not adhere to the OCPs during the shortage	42 (12.5)
Buy the preferred OCPs brand from the black market in fresh dollars	31 (9.2)
Skip some doses of the OCPs	27 (8)
Get the preferred OCPs brand from charities	25 (7.4)

^a^ As multiple responses were given, numbers do not add up to 336

### Impact of the OCPs shortage on women’s quality of life

Participants were inquired about their experienced level of stress when they did not find their preferred OCPs in the Lebanese market. The mean level of stress reported was high 3.92 ± 0.98. “I am very worried about my menstrual period regularity. Well, I’m becoming more stressed and I’m experiencing hair loss due to hormonal imbalance,” Participant 28 stated. “Not finding my medication is messing up my treatment, I’m worried that if I did not take my medication regularly, I might face infertility in the future, this is making me very angry and nervous,” Participant 75. “I am very terrified, as I have to search numerous pharmacies and I am worried that I might experience again severe menstrual bleeding and pain,” Participant 307 reported.

 Moreover, participants reported experiencing several psychological and physiological disturbances during the OCPs shortage including mood disturbances (176, 52.3%), deregulation of menses (167, 49.7%), menstrual pain (71, 21.1%), headache (66, 19.6%), weight gain (66, 19.6%), and acne (53, 15.7%). Furthermore, some participants reported experiencing hirsutism (42, 12.5%) as they were not adherent to their OCPs. “My body became very disturbed since I discontinued taking my medication for PCOS. I’m experiencing uncomfortable body and facial hair growth, weight gain, acne, severe headache, and constipation. This is annoying me,” Participant 10. Remarkably, 32 women disclosed the occurrence of unplanned pregnancies during the OCPs shortage period (9.5%), where 24 of them (75%) declared intentional abortion, and the remaining reported spontaneous abortion. “I experienced an unintended pregnancy as I was not adherent to the medication, but I had to get an abortion since my husband and I cannot afford to raise a newborn,” Participant 25 said.

### Impact of the increased OCPs prices

More than one-third of the participants (165, 37.5%) reported being afflicted by the increased OCPs prices. Of these participants, 35 reported asking for a cheaper alternative brand (21.2%). “The medications are very expensive and yet out of stock. Here in Lebanon, we are living more and more in unlivable conditions,” Participant 261 replied. Notably, some participants revealed that they were skipping some OCPs doses as they could not afford the increased price (24, 14.5%), while a few participants were asking their relatives to get it aboard (15, 9.1%) or were obtaining free samples from non-governmental dispensaries (8, 4.8%). “I am getting an alternative brand for birth control as free samples from Makhzoumi Foundation since my preferred brand has become very expensive,” Participant 145 reported.

### Reasons for avoiding pregnancy and measures to be taken in case of unplanned pregnancies

Participants who were taking OCPs for birth control were inquired about their reasons for avoiding pregnancy (177, 40.5%). Almost half of them reported being satisfied with the number of children they already have (99, 55.9%), while some participants preferred to avoid pregnancy due to the current economic situation (74, 41.8%), and due to the social and political instability in the country (47, 26.6%). Other participants noted that they were not willing to have a child since they do not want to handle extra responsibilities (33, 18.6%), they do not have enough time to raise a child (21, 11.9%), or their partners do not want to have a child (17. 9.5%). A few participants explicitly disclosed that they were not willing to get pregnant since they are still unmarried (6, 3.37%).

The mean level of stress associated with the risk of unplanned pregnancy among the participants was 3.97 ± 1.05. The women were questioned about what to do in case of an unplanned pregnancy were one-fourth of them disclosed that they would consider abortion (44, 24.9%). “My kids are still toddlers, and my husband’s salary is low. We cannot afford to raise a new child, so I might consider getting an abortion in case of unplanned pregnancy,” Participant 331 answered. On the other hand, 133 participants reported that they would maintain the unplanned pregnancy (75.1%). “My husband forbids me from getting an abortion since in Islam abortion is forbidden,” Participant 347 stated.

### Alternative contraceptive methods used during the OCPs shortage and their impact on the participants’ sexual life

Out of the 177 participants using OCPs for contraception purposes, almost half of them reported using condoms as an alternative method during the OCPs shortage (79, 44.6%). In addition, more than half of the participants (98 out of 177, 55.3%) declared using traditional contraceptive methods such as ejaculation withdrawal (34, 19.2%), sexual abstinence, (29, 16.2%), sexual avoidance during ovulation days (29, 16.2%), and using emergency contraceptive pills after having sexual intercourse (6, 3.4%).

Almost half of those participants using OCPs for contraception noted that the OCPs shortage has decreased the frequency of sexual intercourse (86, 48.6%). This has impacted their quality of life in such a way that it has led to conflicts with their partners (40, 46%), mood changes (37, 42.5%), anxiety (33, 37.9%), decreased libido (23, 26.7%), and reduced self-esteem (13, 14.9%). “My relationship with my husband is deteriorating as I am psychologically distressed, and I’m experiencing a lot of tension lately,” Participant 30 replied. “My sexual desire has decreased, and my husband has become more nervous since I am not satisfying his sexual needs,” Participant 65 responded.

## Discussion

The current study revealed that a significant proportion of consumers were not able to secure their favorite OCPs commodity from the Lebanese market for a considerable period. The sparse availability of OCPs deprives females of their right to access sexual and reproductive health care. In addition, failure to provide an adequate stock of OCPs threatens the reproductive health of women and families.

Several countries have also reported a shortage of hormonal contraceptives during the COVID-19 pandemic, including the United Kingdom, South Africa, and Australia, where women were frustrated and forced to find an alternative birth control method [35, 40–42]. These countries announced shortages in one or two contraceptive commodities due to worldwide manufacturing constraints and the shortfall in the supply of hormonal contraceptives during the pandemic [35, 40–42]. However, the situation here in Lebanon is more complicated since most of the OCP brands were unavailable for a significant period, as reported by the study participants. A large number of consumers reported running out of supply, being forced to visit several pharmacies to find their brand, and becoming more distressed to find alternative products. Switching to an alternative OCP brand might waste women’s time in “trial and error” to find suitable alternative pills, which in turn will influence their adherence [43].

Conspicuously, one-third of the participants reported stockpiling their OCP brand during the current crisis. This raises concerns about the inequitable distribution and dispensing of OCPs among consumers. It is worth mentioning that Lebanese community pharmacists have no access to patients’ electronic health records, which hinders them from allocating healthcare resources and balancing community needs. Thus, it is recommended to implement a universal electronic healthcare system that links the community pharmacies with different healthcare centers and the Lebanese Ministry of Public Health, which should aid in improving healthcare provision, facilitating traceability of medication dispensing, and hence ensuring fair drug dispensing and limiting drug monopoly.

Despite the fact that the OCPs shortage does not directly impose an immediate risk on women’s health, it has a potential impact on females’ physiological, psychological and reproductive well-being, in addition to increasing the risk of unexpected pregnancies, which may lead to serious health sequelae and subsequent abortions. Most of the study participants who receive OCPs for contraceptive purposes expressed the high level of stress experienced from the risk of having unplanned pregnancies. Notably, one-quarter of the participants disclosed their intention to get an abortion if they have an unexpected pregnancy. In 2021, England and Wales have reported the highest abortion rate, since 1967, among their residents (18.6/1,000 women) [44]. Although there are several reasons behind this, the occurrence of unwanted pregnancies due to nonadherence to contraceptives or the difficulty in obtaining the usual contraceptives remain potentially preventable causes. According to the United Nations, “the prevalence of contraceptive use and unmet need for family planning are key indicators for measuring improvements in access to reproductive health” [43].

More than half of the study participants using OCPs for pregnancy prevention reported adopting alternative traditional contraceptive methods during the shortage. The traditional contraceptive methods utilized included the calendar or rhythm method, sexual abstinence, and coitus interruptus (withdrawal method). The use of traditional contraceptive methods in the current study was much higher than that of other developing countries, which was already reported by the United Nations in 2020 [43]. For instance, the adoption of traditional contraception was 17% in Lebanon and Bahrain, 15% in Syria, 10% in Jordan, 5% in the United Arab Emirates and Kuwait, and 2% in Saudi Arabia [45]. It is worth mentioning that utilizing contemporary traditional contraceptive methods is less effective than modern hormonal contraception [46–48].

The medication shortage predicament in Lebanon is also aggravated by the rising medication prices, which imposes a serious and persistent challenge on the healthcare system, especially after lifting the medication subsidy [34]. Almost one-third of the study participants reported being affected by the rising OCPs’ prices, where some of them could not afford the price and were forced to search for cheaper alternative brands or to discontinue taking OCPs. Such behavior may threaten women’s reproductive health and expose them to unplanned pregnancies [49, 50]. Therefore, a judicious strategy should be implemented in Lebanon to rein in out-of-control drug prices and address the drug shortages that are putting a tremendous strain on the healthcare system and patient care. Health policymakers should secure a stable source of medications by investing in national pharmaceutical manufacturing, which can be implemented by modifying the subsidy system to support local pharmaceutical production [34]. Moreover, the availability of cheaper, bioequivalent generics may lower the burden on the community [51].

### Limitations of the study

This study was proactive in exploring the consequences of the OCPs shortage in Lebanon. However, it was associated with several limitations. First, this study has only recruited women encountered in community pharmacies. This would introduce selection bias since other OCPs utilizers would have either previously stockpiled their OCPs brand, not visited the community pharmacies out of desperation, or adopted an alternative contraceptive method. However, we have prolonged the duration of the data collection for five months to achieve a representative number of participants and to reflect the women’s actual experience. Similarly, those who were already unintentionally pregnant due to the lack of OCPs may not have visited the pharmacies to obtain them. Therefore, the results may have underestimated the prevalence of unplanned pregnancies and their consequences such as abortion. Second, the interview-based nature of this study is a potential weakness due to the possible introduction of social-desirability bias. For instance, there was an underrepresentation of illiterate and single females, especially that around two-thirds of the study participants had graduate studies. This can be justified by the fact that this study tackles a culturally sensitive topic, in which the participation of uneducated and unmarried females might be inappropriate. Moreover, participants might not report the actual purpose of using OCPs, especially since the use of OCPs among single females in conservative societies with prevailing Islamic religion is looked at as taboo because sexual activity outside marriage is prohibited. Furthermore, participants might not reflect their actual intention to deal with an unplanned pregnancy, particularly since abortion in Lebanon is illegal and impious. Third, this study did not utilize a well-validated screening tool for stress. Finally, the predictors for adopting traditional contraceptive methods were not investigated. However, this is an exploratory study that aimed to provide a transparent and holistic image of the current situation without thoroughly examining the causality.

## Conclusions

The current economic crisis in Lebanon has worsened the medication shortage and led to inflation of prices. The incidence of OCPs shortage was significantly high, where the most coping strategy adopted in response to this shortage was the use of traditional contraceptive methods. The related shortage and increased prices of OCPs have exposed women to various undesirable consequences including unplanned pregnancy, dysregulation of menses, stress and mood disturbances, acne, hirsutism, and decreased sexual libido. In addition, the hyperinflation of OCPs prices has considerably affected the consumers’ ability to secure their OCPs. Therefore, there is an urgent need to bring the attention of healthcare authorities to support the national pharmaceutical industry in manufacturing affordable OCPs generics to meet women’s reproductive health demands.

## Data Availability

The dataset presented in this article is available only upon reasonable request since it contains confidential information. Requests to access the datasets should be directed to the first author (r.itani@bau.edu.lb).

## Abbreviations

FDA: Food and drug administration
IOC: Item objective congruence
NGO: Non-governmental organization
OCP: Oral contraceptive pill
PCOS: Polycystic ovarian syndrome
PI: Principal investigator
SD: Standard deviation
SPSS®: The Statistical Package for the Social Science
